# Controlling item difficulty for automatic vocabulary question generation

**DOI:** 10.1186/s41039-017-0065-5

**Published:** 2017-12-19

**Authors:** Yuni Susanti, Takenobu Tokunaga, Hitoshi Nishikawa, Hiroyuki Obari

**Affiliations:** 1 Department of Computer Science (W8E-6F), Tokyo Institute of Technology, 2-12-1 Oookayama, Meguro-ku, Tokyo, 152-8552 Japan; 2 College of Economics, Aoyama Gakuin University, Tokyo, Japan

**Keywords:** Item difficulty, English vocabulary question, Automatic question generation, Multiple-choice question

## Abstract

The present study investigates the best factor for controlling the item difficulty of multiple-choice English vocabulary questions generated by an automatic question generation system. Three factors are considered for controlling item difficulty: (1) reading passage difficulty, (2) semantic similarity between the correct answer and distractors, and (3) the distractor word difficulty level. An experiment was conducted by administering machine-generated items to three groups of English learners. The groups were determined based on their standardised English test scores. In total, 120 items, generated using combinations of the above three factors, were tested. The results reveal that the distractor word difficulty level had the greatest impact on item difficulty, but this tendency changed depending on the proficiency of the test takers. These results will be of use when implementing a fully automatic system for administrating tests.

## Introduction

Research on computer-assisted language testing mainly focuses on areas related to the delivery of the test, e.g. computerised adaptive testing (CAT), and other areas such as automatic test scoring, response analysis, and item generation. Research on designing the difficulty of test items, however, is still relatively rare despite its significance in the research areas mentioned above. For instance, the ability to design item difficulty automatically would be an advantage for the task of automatic item generation, so that the generated items are of suitable difficulty for a particular group of test takers. To illustrate, a test composed of only too easy or too difficult items for particular groups would not be very informative about the ability of the test takers in those groups; in other words, the test would fail to discriminate the test taker’s ability on the subject. This is especially important in tests for admission and class placement.

In CAT, item difficulty also plays an important role. It is one of the parameters^1^ that decides which item(s) to present and is used for estimating a test taker’s proficiency, which are the characteristics of an adaptive test. An adaptive test is administered by adjusting the items according to each test taker’s ability, which is estimated during the test. Thus, the item difficulty should be known before the item is presented to the test taker. In the current CAT, item parameters, including item difficulty, are commonly estimated using test takers’ responses in a process called item calibration. Item calibration is done either using the existing test data or by conducting an item pretest. For the CAT algorithm to be able to select items tailored to test taker’s ability, many items of various difficulty levels are needed. This results in considerable costs for item development, pretesting, and item calibration (Veldkamp and Matteucci 2013). The test taker’s motivation in an item pretest could pose another problem as well, because it could lead to invalid calibration if the test taker attaches no importance to their results (Davey and Pitoniak 2006). For instance, if the test takers know that the pretest does not reflect on their grade, they might not work seriously. In addition, conducting an item pretest poses the risk of exposing the item before it is used in a real test.

Studies related to the item difficulty of language test are directed more toward prediction than controlling the item difficulty. These are fundamentally different tasks because the former concerns how difficult an item is, while the latter is concerned with how to create items of various levels of difficulty. Earlier work on predicting item difficulty has been done on reading and listening comprehension tests using multiple regression combined with regression tree analysis (Rupp et al. 2001) and artificial neural networks (Boldt and Freedle 1996; Perkins et al. 1995). More recently, Loukina et al. (2016) conducted a study to investigate the extent to which textual properties of a text affect the difficulty of listening items in the English test. Trace et al. (2015) used item and passage characteristics to determine the item difficulty of cloze questions^2^ across the test taker’s nationality and proficiency level. Other studies focused on vocabulary questions, as conducted by Hoshino and Nakagawa (2010), Beinborn et al. (2014), and Susanti et al. (2016). Beinborn et al. (2014) worked on predicting the gap difficulty of the C-test^3^ using a combination of factors such as phonetic difficulty and text complexity, while Hoshino and Nakagawa (2010) and Susanti et al. (2016) investigated factors affecting item difficulty on multiple-choice vocabulary questions.

Unlike this previous research, the aim of the present study is to control item difficulty in the automatic question generation task. Automatic question generation has been proven to be able to generate questions with a quality that is comparable to those made by humans (Susanti et al. 2017). Moreover, they cannot be distinguished from human-made questions (Le and Pinkwart 2015; Susanti et al. 2017). Our long-term goal is to integrate automatic question generation into CATs, where the question items are created on the fly before they are presented to test takers. To our knowledge, studies on controlling item difficulty are scarce. Cheng (2010) mentioned that there are four elements of an item in which difficulty may reside: the content to be assessed, the stimulus (the accompanying things that come with the question, e.g. tables or diagrams), the task to be performed, and the expected response. Therefore, he suggested controlling the item difficulty by varying these four elements. Hohensinn and Kubinger (2009) investigated item difficulty control by changing the response format in math items and found that the different response formats measure the same ability but bias the item difficulty.

In this study, we propose a method that controls the item difficulty using three predetermined factors related to the question components: (1) reading passage difficulty (RPD), (2) semantic similarity between the correct answer and distractors (SIM), and (3) distractor word difficulty level (DWD). The first factor is important because the question type in our research is a question that asks for the closest meaning of a word in a particular reading passage; so if the reading passage is difficult, this might make the question difficult as well. The second factor is also important because if the question options are similar, it is harder for the test taker to choose the correct answer. The distractor word difficulty has also been shown to affect the difficulty of an item (Susanti et al. 2016), so we consider this factor too. The three factors are explained in detail in the later section. We conducted an experiment on real learners and analysed the collected data with respect to the following research questions:

1. Can the item difficulty be controlled by the investigated factors?
2. Which among the investigated factors contributes the most to the item difficulty?
3. How do these factors affect the item difficulty across test takers with different proficiencies?

The results reveal that the DWD level had the greatest impact on item difficulty, but this tendency changed depending on the proficiency of the test takers.

The remainder of this paper is organised as follows. We next present an overview of the automatic question generation system (“Automatic question generation system” section), followed by our proposed method for controlling item difficulty (“Method: controlling item difficultyMethod: controllingitem difficulty” section). Then, we present the evaluation of our method (“Evaluation designEvaluationdesign” section) and its results and a discussion (“Results and discussion” section). Finally, we conclude the paper and provide future research directions (“Conclusion” section).

## Automatic question generation system

Research on automatic question generation dates back to the late twentieth century, when Wolfe (1976) described an experimental computer-based educational system called AUTOQUEST. The aim of AUTOQUEST is to assist the self-study of written text, and its investigation is one of the initial studies in the field of automatic question generation. As Wolfe claims, studying using questions could potentially enhance a learner’s reading comprehension skill, but a considerable manual effort is needed to prepare those questions. Since then, there have been a substantial number of studies on automatic question generation, particularly for the English language learning/test purposes (Araki et al. 2016; Brown et al. 2005; Lin et al. 2007; Smith et al. 2010; Susanti et al. 2015). Multiple-choice questions in particular have received extra attention because they are used in standardised English proficiency tests such as TOEFL, TOEIC, and IELTS. For instance, Hoshino and Nakagawa (2005) developed a real-time system that generates multiple-choice questions on English grammar and vocabulary from online news articles. More recently, Susanti et al. (2015) generated the multiple-choice English vocabulary questions currently used in TOEFL, while Satria and Tokunaga (2017) worked on the TOEFL English pronoun reference questions. Araki et al. (2016) introduced a method to generate multiple-choice open-ended questions for reading comprehension. Other studies worked on cloze, or fill-in-the-blank questions, paying more attention to generating more distracting distractors (Sakaguchi et al. 2013; Zesch and Melamud 2014).

**Fig. 1:**
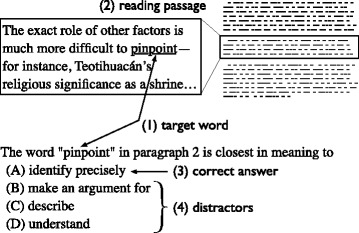
Four components in a multiple-choice question asking for closest-in-meaning of a word (source: TOEFL iBT question from past test, taken from the official website, http://www.ets.org)

The aim of the present study is to control the item difficulty of the questions generated by an automatic question generation system. We implemented an automatic question generation system to generate the closest-in-meaning vocabulary questions, which are modelled after the vocabulary questions used in TOEFL, as proposed by Susanti et al. (2015). Figure 1 shows an example of the vocabulary questions used in this study.

As shown in Fig. 1, a question is composed of four components: (1) a target word, which is the word being tested in the question, (2) a reading passage, in which the target word appears, (3) the correct answer, and (4) three distractors, or incorrect options. In this type of question, the test takers are instructed to find the word with the closest meaning to the target word when it is used in a particular context provided by the reading passage. Closest-in-meaning vocabulary questions intend to measure the ability of the test takers to understand a word when it is used in a particular context. There is only one correct answer among the four options.

Given a target word and one of its word senses (meanings) as the inputs, the process of generating a question starts with retrieving a reading passage containing the target word with the given sense from Web news articles on the Internet. The retrieved reading passage and a lexical dictionary are utilised to generate the remaining question components (Susanti et al. 2015). In the automatic vocabulary question generation, the item difficulty can be controlled during the process of generating each component. For instance, the system retrieves an easy reading passage for an easy item, while it retrieves a difficult reading passage for a difficult item. We explain the detailed method for controlling the item difficulty in the automatic question generation in the next section.

## Method: controlling item difficulty

This section describes our proposed method for controlling the item difficulty in the automatic vocabulary question generation system described in the previous section.

In classical test theory (CTT), item difficulty or the difficulty index is defined as the proportion of test takers who correctly answered a question item; thus item difficulty can be estimated only after administering the item to the test takers. The item difficulty can then be used to determine whether a certain item is appropriate (not too easy nor too difficult) for a group with a certain ability.

However, administering items using a pretest is costly, and there is a risk of exposing the items before they are used for a real test. Moreover, a considerable number of test taker’s responses are required to obtain a reliable estimate of item difficulty (Loukina et al. 2016). Hence, the aim of the present study is to control item difficulty intrinsically, i.e. control the item difficulty through the characteristics of the question components (reading passage, correct answer, and distractor, as illustrated in Fig. 1) during the item generation process.

Hoshino (2013) analysed the relationship between different types of distractors and difficulty of question items in the multiple-choice vocabulary cloze test. Three sets of question items were prepared with various types of distractors based on their relation to the other components of the question: (a) distractors with a paradigmatic relationship with the correct answer, (b) distractors with a syntagmatic relationship with the context sentence, and (c) those with no relationship with either the correct answer or the context sentence. Their result showed that question items with type (a) and (b) distractors were more difficult than those with type (c) distractors.

More recently, Susanti et al. (2016) conducted an investigation of several potential factors affecting item difficulty in the vocabulary questions used in TOEFL. All investigated factors are related to the components of the vocabulary questions, as presented in Fig. 1. Their study revealed that (1) the word difficulty level of the question components contributed up to 60% of the item difficulty and (2) distractors had the greatest impact on item difficulty.

Both Hoshino (2013) and Susanti et al. (2016) indicated that the distractor played a critical role in the difficulty of multiple-choice vocabulary questions. Based on their results, the following three factors are considered in the present study: (1) RPD, (2) SIM, and (3) DWD. For each of these factors, we define two levels, as shown in Table 1.

**Table 1:** Investigated factors

ID	Factor	Level	
RPD	Reading passage difficulty	Low	High
SIM	Semantic similarity between correct answer and distractor	Low	High
DWD	Distractor word difficulty level	Low	High

The following is a detailed explanation of how we implement these three factors in the automatic question generation system.

### Reading passage difficulty (RPD)

The first factor investigated for its effect on item difficulty is the reading passage difficulty. In the vocabulary questions used in the present study, the test takers are expected to find the word with the closest meaning of the target word when it is used in the given context (reading passage). Hence, the difficulty, or readability, of the reading passage should affect the item difficulty. Readability is defined as the level of ease at which text material can be understood by a particular reader who is reading that text for a specific purpose (Pikulski 2002).

According to Susanti et al. (2016), the word difficulty of the question components contributes to the difficulty of question items by up to 60%. Because we adopt only two levels of reading passage difficulty, two fixed sources for retrieving reading passages were used: the *Times in Plain English* http://www.thetimesinplainenglish.com/ for low (easy) reading passages and the *New York Times* http://nytimes.com/ for high (difficult) reading passages^4^. We applied three readability measures to calculate the difficulty of the two reading passage sources, namely the Flesch-Kincaid Grade Level, Flesch-Kincaid Reading Ease (Kincaid et al. 1975) and Dale-Chall readability formula (Chall and Dale 1995), which are based on the sentence length and word difficulty.

We sampled 20 articles from those two websites and calculated the difficulty using the three readability measures. The average readability scores for the 20 articles are shown in Table 2 5. For the Flesch-Kincaid Grade Level and Dale-Chall readability formulas, a higher score means a more difficult text, while the Flesch-Kincaid Reading Ease score is the opposite. For example, a value of 4.98 under the *Times in Plain English* column in Table 2 means that on average, 4.98th US grade level (5th grade) students understand the articles. The table shows that in our sample, the articles from *New York Times* are more difficult than the articles from *Times in Plain English* regarding all readability measures (the differences are significant with *p* value <.01). Thus, we used the two sites as sources for the low and high levels of reading passage, respectively.

**Table 2:** Readability measures of two sources

Readability measure	Source	
	*Times in Plain English*	*New York Times*
Flesch-Kincaid Grade Level	4.98	11.03
Flesch-Kincaid Reading Ease	75.9	52.24
Dale-Chall Formula	6.27	8.1

### Similarity between correct answer and distractors (SIM)

The second factor is the semantic similarity between the correct answer and distractors. Distractors in a multiple-choice question act as a lure to distract the test takers from finding the correct answer. The vocabulary question used in the present study asks for the word with closest meaning to that of the target word; thus, a distracting distractor would be one with a close but different meaning from the correct answer. Hence, the similarity between the correct answer and distractors is considered to be a factor affecting item difficulty.

A vector space model is utilised to calculate the similarity between the correct answer and distractors based on the idea that similar words tend to occur in similar linguistic contexts. In the vector space model, a word is represented by a vector of elements corresponding to the frequency of the co-occurrence words within a certain window in the corpus. To count the frequency, we make use of available corpora in the NLTK Python package^6^.

The distractor candidates are collected from several sources, including the synonyms of the co-occurrence words with the same part-of-speech as the target word in the reading passage, and the sibling and hyponym words of the target word in a lexical taxonomy. These candidates are filtered and the cosine similarity is further used for calculating the word vector similarity between the correct answer and every distractor candidate (Susanti et al. 2015). The three candidates with the lowest similarity are chosen for the low-level distractors, and the three candidates with the highest similarity are chosen as the high-level distractors.

### Distractor word difficulty level (DWD)

The last factor is the word difficulty level of distractors. Distractors in multiple-choice questions have been shown to affect item difficulty (Hoshino 2013; Susanti et al. 2016). The JACET8000 list of words (Ishikawa et al. 2003) is used to determine the word difficulty level in this work, as it is a list designed for Japanese English learners, who were the participants in the experiment.

When generating distractors, the system eliminates irrelevant distractor candidates following several requirements proposed in the past research (Susanti et al. 2015). For instance, we should avoid pairs of synonyms as distractors because they can be easily ruled out by the test takers (Heaton 1989). These filtered distractor candidates are then ranked based on the semantic similarity and further divided into two levels of distractor candidates: low and high, as explained in the “Similarity between correct answer and distractors (SIM)Similarity between correct answer anddistractors (SIM)” section. Next, the word difficulty level is retrieved for each distractor candidate based on the JACET8000. The three candidates with the lowest level are further adopted as the low (easy)-level distractors, and the three highest level candidates are adopted as the high (difficult)-level distractors. If a distractor is composed of multiple words, we calculate the average of the JACET8000 word difficulty levels of each word after removing the stopwords^7^.

## Evaluation design

The main purpose of this evaluation is to investigate the impact of the three potential factors on the item difficulty of the vocabulary questions. We asked university students to take a test composed of machine-generated vocabulary questions that were generated with different combinations of factor levels (low and high).

### Question data

We created all eight possible combinations of the three factors with two levels each, as shown in Table 3. Question items were generated that conformed to each combination. Note that each question item asked for a different target word at the same level of word difficulty based on JACET8000, which uses an eight-level system in which level 1 denotes the easiest words (most frequently used words). The target words were selected from the list at JACET8000 difficulty level 4 after consulting with the English teacher in charge of the class that our participants attend because he is familiar with their English proficiency.

**Table 3:** Combinations of three factors

Combination	Factor		
	RPD	SIM	DWD
LLL	Low	Low	Low
LLH	Low	Low	High
LHL	Low	High	Low
LHH	Low	High	High
HLL	High	Low	Low
HLH	High	Low	High
HHL	High	High	Low
HHH	High	High	High

We prepared 15 question items for each combination in Table 3, generating 120 question items in total. Note that we used 120 different target words for these question items. We divided the 120 items into three question sets, taking into account the balance of the combinations and parts-of-speech of the target words. One question set consisted of 40 question items, with five items for each combination. We conducted a pilot study and determined that 40 is a reasonable number of items to assign test takers in a single session.

### Participants

A total of 88 Japanese university undergraduate students participated in the experiment. We divided them into three groups^8^, keeping a close distribution (mean and standard deviation) of their TOEIC scores within the groups and administered a different question set for each student group. Table 4 shows the assignment of the question sets to the student groups.

**Table 4:** Configuration of the question sets

Student group	Question set	Target word
G1	QS. A	TW 1-40
G2	QS. B	TW 41-80
G3	QS. C	TW 81-120

### Experimental procedure

The experiment was conducted as an online test. The participant took the test using a computer. The experiment comprised three sessions, in each of which one of the three groups worked on the assigned question set shown in Table 4. A session was 30 min long. All participants in each group worked on the question set together in the same classroom.

## Results and discussion

In total, 10,560 responses for all question items (88 students worked on 120 question items) were collected in the experiment. Table 5 shows the average score for each student group, together with the students’ TOEIC scores. The test score of a test taker was calculated by dividing the number of their correct responses by the total number of questions in the question set, i.e. 40. Pearson correlation coefficients^9^ were calculated between the test scores and the TOEIC scores of the students in each group. The average of the TOEIC scores and the test scores for each group are not largely different; in addition, the two scores are moderately correlated for all groups, as shown in Table 5, except for group G3. We investigated this phenomenon further to find possible reasons for it. We checked the items in the question sets administered to G3, and we did not find any peculiar characteristics or technical mistakes that would make the performance of G3 distinct from those of G1 and G2. We can say that there was no linear correlation between the test taker’s scores on the test and their TOEIC scores. There could be several unlikely cases that were probably the cause of this, e.g. test takers with low or high TOEIC scores could both have attained similar scores on the test or test takers with high TOEIC scores could have gotten low test scores and vice versa.

The discussion in this paper addresses the three research questions posed in the “Introduction” section:

1. Can the item difficulty be controlled by the investigated factors?
2. Which among the investigated factors contributes the most to the item difficulty?
3. How do these factors affect the item difficulty across test takers with different proficiencies?

Each question is dealt with in the subsequent sections.

**Table 5:** Test scores vs. TOEIC score for each student group

	G1	G2	G3
Number of students	31	28	29
TOEIC avg	606.33	594.07	596.42
	(SD: 166.11)	(SD: 170.81)	(SD: 178.37)
Test score avg	.496	.471	.581
	(SD:.1)	(SD:.1)	(SD:.09)
TOEIC-Test score corr.	.693	.612	−.09

### Research question 1: can the item difficulty be controlled by the investigated factors?

To answer the first research question, we first look at the average difficulty for each combination. If the average difficulty for each combination is different, it could mean that we can control the item difficulty by varying the factors.

#### Estimating the item difficulty

There are several ways of estimating item difficulty from the test taker’s responses. In test theory, such as CTT and item response theory (IRT), the difficulty is defined as the likelihood of correct responses, not as the perceived difficulty or necessary amount of effort (DeMars 2010).

According to CTT, item difficulty, more commonly referred to as the difficulty index (*P*), is simply the proportion of test takers who correctly answer the item. Suppose an item is correctly answered by eight out of ten test takers. Then the CTT difficulty index would be 0.8 (8/10). Hence, the item difficulty ranges from 0 to 1, with a higher value suggesting an easier item.

DeMars (2010) explained that in IRT, the item difficulty (*b*) represents the proficiency of the test takers, half of which are expected to answer the item correctly. For instance, if *b* = 0.2, about 50% of test takers with proficiency = 0.2 would answer the item correctly. In contrast to CTT, a higher *b* value indicates a more difficult item.

We calculated the estimated item difficulty of all question items using both CTT and IRT (using R^10^ software and the lazyIRT package^11^). One question item was removed from the IRT analysis because no test taker correctly answered it. The descriptive statistics for the estimated item difficulties are presented in Table 6. We found that the difficulties estimated by CTT and IRT are strongly correlated (*r* =.80). Hence, for further analysis, we use only the CTT difficulty (*P*).

**Table 6:** Descriptive statistics for the estimated item difficulty

	*P*(CTT)			*b*(IRT)		
	QS. A	QS. B	QS. C	QS. A	QS. B	QS. C
*n*	40	40	40	39	40	40
x̄	.491	.529	.419	.296	1.278	−1.720
sd	.213	.228	.275	5.523	3.819	6.987
max	.968	.964	.931	20	10.805	15.588
min	.065	.036	.034	−15.974	−5.922	−20

#### Analysis of variance on combinations

**Fig. 2:**
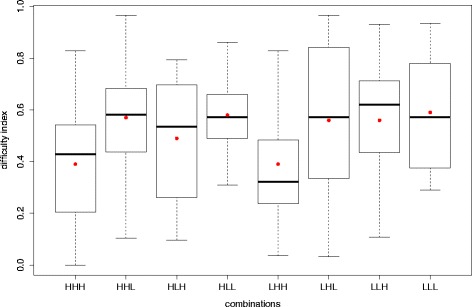
Box plot for eight combinations

Figure 2 shows the boxplot of the average difficulty index *P* for each combination. The boxplot shows that the means (red circles) are different for each combination. However, the difference between combinations varies greatly according to the combinations. For instance, the mean difference between HHH and LLL is greater than LLH and LHL, while LLL and LLH have large overlap in difficulty. Hence, these differences in means could have come about by chance. We performed a one-way analysis of variance (ANOVA) on the combinations to see if the differences between them are statistically significant. We subsequently looked at the *p* value of the ANOVA results to determine to what extent the differences between the means are significant.

According to Levshina (2015), the following assumptions should be met before performing a one-way ANOVA:

- Independence of observations. No participant in the experiment should belong to more than one group.
- The dependent variable is at least interval-scaled.
- Each sample is drawn from a normally distributed population and/or the sample sizes are equal. It is difficult to evaluate the normality because the number of our data is small. However, the sample sizes for each combination are equal (15 items for each combination). Normality is also tested using the Shapiro-Wilk^12^ normality test, and the result shows that none of the *p* values of the combinations is smaller than.01. Therefore, we do not have reasons to believe that the assumption of normality is not met.
- The variance is homogeneous. The variance is tested using the Levene test^13^. The null hypothesis of the Levene test is that the combinations have equal variances. The result shows that the *p* value is greater than.01, so the null hypothesis of equal variances cannot be rejected.

We performed the ANOVA on (1) the eight combinations shown in Table 3 and (2) four regrouped combinations, as explained below.

**Eight combinations** The one-way ANOVA was performed on the eight combinations (Table 3), yielding in a quite high *p* value (.0913). This indicates that the mean differences between the eight combinations are not statistically significant at a significance level of.05.

**Fig. 3:**
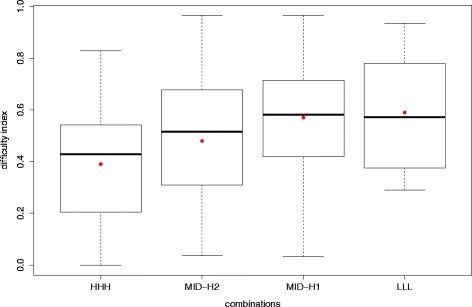
Box plot for four regrouped combinations

**Four regrouped combinations** We could not find significant differences among the eight combinations. Because of the limited number of participants, we could only administer 15 items for each of the eight combinations. We conducted the ANOVA on a coarser set of combinations as well. We reduced the combinations into four groups based on the number of “high” factors: (1) LLL, (2) MID-H1 (LHL + LLH + HLL), (3) MID-H2 (LHH + HHL + HLH), and (4) HHH. The result of ANOVA shows that the difficulty differences between these four new groups are statistically significant (*p* value <.05). This indicates that setting the factors to high or low indeed influences the item difficulty; to be more concrete, the items with more “high” factors are more difficult than those with fewer “high” factors. Therefore, item difficulty can indeed be controlled by varying the investigated factors, which answers the first research question. Figure 3 shows the box plot of the reduced combinations.

### Research question 2: contribution of each factors

The following analysis presents the contribution of each factor toward explaining item difficulty.

A three-way ANOVA was performed to determine if a three-way interaction effect exists among RPD, SIM, and DWD for explaining item difficulty, as well as to understand which factor contributes the most to item difficulty. The ANOVA result shows that there is no significant three-way interaction (*p* value >.050), meaning that the investigated factors are independent of each other. The result also revealed that DWD has the biggest influence on item difficulty, with a *p* value of.008, followed by SIM (*p* value =.080), and RPD has the least influence on item difficulty (*p* value =.710).

**Main effect** In addition to the three-way interaction, there are three main effects that can be observed for each of the three factors. For example, the main effect of RPD is the difference between the means of the item difficulty for the two levels of RPD (RPD.high and RPD.low), ignoring the other two factors. The means for a three-factor experiment are often displayed in the form shown in Table 7. From the table, we can calculate the main effect of each factor by subtracting the means of the two levels, as presented in the following.

**Table 7:** Item difficulty means values

		DWD.low	DWD.high	Row mean
RPD.low	SIM.low	.590	.557	.573
	SIM.high	.559	.392	.475
	Mean	.574	.474	.524
RPD.high	SIM.low	.583	.490	.536
	SIM.high	.571	.388	.479
	Mean	.577	.439	.507
	Column Mean	.575	.456

- RPD. (high=.507−low=.524)=−.017
- SIM. (high = (.475+.479)/2 =.477) − (low = (.573 +.536)/2 = .555) = −.078
- DWD. (high=.456−low=.575)=−.119

The large difference of the main effect above means that the factor affects on discerning between the high and low level, leading to a large impact on item difficulty. Among the three factors, the effect of DWD on item difficulty is statistically significant at a level of.05. In addition, the participants were more affected by the question options factor (DWD and SIM) than the reading passage factor (RPD). The test takers likely only looked at the target word in the passage and the question options to determine the correct answer. They did not read the whole reading passage or did read only the local context around the target word. This finding suggests that in the multiple-choice vocabulary questions, the question options, especially the distractors, are the most important factors for determining the question difficulty.

### Research question 3: item difficulty in proficiency-based groups

The previous research question asked the impact of each factor on the overall item difficulty. The research question 3 further asks how the impact differs across different proficiency groups. We divided the test takers into three groups based on their TOEIC scores: high proficiency group (≥ 691), middle proficiency group (496 ∼690), and low proficiency group (≤ 495). The TOEIC scores of two test takers were not available, so we excluded them from the following analysis. Table 8 shows the descriptive statistics for the three groups. We treat the TOEIC scores as the true proficiency scores. We carried out the three-way ANOVA test on each group to determine how the impact of the three factors on the item difficulty differs for each of the proficiency-based groups.

**Table 8:** Descriptive statistics of item difficulty *P* for proficiency-based groups

	Low group	Middle group	High group
*n* student	23	36	27
x̄	.456	.507	.562
sd	.278	.262	.297
max	1	1	1
min	0	0	0

**High proficiency group** As discussed in the previous sections, the general tendency for all test takers is that the DWD affects the estimated item difficulty the most. However, this tendency is not retained in the high proficiency group, where SIM has the most significant influence on the item difficulty (*p* value =.005), followed by DWD (*p* value =.06), and RPD (*p* value =.95). This finding suggests that the high proficiency students are more likely to get confused when the correct answer and distractors have a similar meaning to each other. Moreover, in this group, RPD does not significantly affect the estimated item difficulty. This suggests that the students probably did not read the reading passage to answer the questions in the experiment; they were probably aware of the target word’s meaning regardless of its context.

**Middle proficiency group** The general tendency is retained in the middle proficiency group. DWD affects the item difficulty the most (*p* value =.001), followed by SIM (*p* value =.065) and RPD (*p* value =.764). Again, RPD has the least impact on item difficulty, suggesting that the test takers in the middle proficiency group probably did not read the reading passage. The general tendency might be influenced by the tendency of the middle group because the majority of the test takers belong to this group (36 out of 88 test takers).

**Fig. 4:**
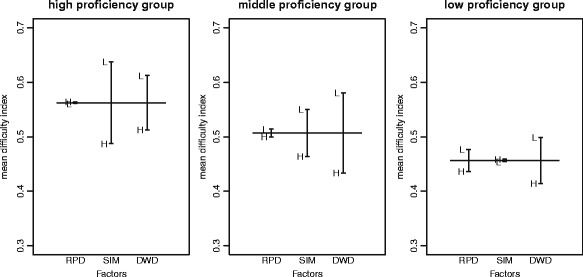
Differences of mean difficulty index across proficiency-based groups

**Low proficiency group** In contrast to the tendency of the middle and high proficiency groups, the test takers in the low proficiency group found the vocabulary items to be more difficult when the DWD was high. The item difficulty is furthermore affected by RPD then followed by SIM. However, the *p* values for all three factors are quite high (.097,.436, and.927, respectively). The SIM factor has the least effect on item difficulty for the low proficiency group, indicating that the test takers in this group might not recognise the case where the correct answer and distractors have similar meaning. Instead, the other two factors that can be recognised by the test takers, i.e. DWD and RPD, affect the difficulty for them. The tendencies for all three groups are illustrated in Fig. 4.

To sum up, the factors affecting item difficulty are different depending on the proficiency of the test takers. For instance, in our experiment, the SIM factor has the greatest impact on the item difficulty for the high proficiency group of test takers while the same factor has the least impact on the low proficiency group. Thus, to design the item difficulty, item writers must consider the proficiency of the test takers.

## Conclusion

The present study investigated how item difficulty can be controlled by the three predetermined factors in an automatic vocabulary question generation system. The three factors are (1) reading passage difficulty (RPD), (2) similarity between the correct answer and distractors (SIM), and (3) distractor word difficulty level (DWD). The experiment was conducted using 120 question items automatically generated by the system and 88 non-native English learners.

We analysed the collected data against to answer three research questions: (1) whether the item difficulty can be controlled using the investigated factors, (2) which factor contributes the most to item difficulty and, (3) how these factors affect the item difficulty across test takers with different proficiency. We first estimated the item difficulty for each item using both CTT and IRT. The estimated item difficulties from both theories correlated quite highly (*r* =.80); hence, for further analysis, we used CTT. The analysis revealed the following results.

We first investigated if the item difficulty could be controlled by the automatic question generation system using the three factors. Each of the three factors was set to two levels, high (difficult) and low (easy). The one-way ANOVA was performed to test the significance of the differences of the mean item difficulty among combinations of the three factors. We prepared two sets of combinations: (1) eight combinations and (2) four regrouped combinations. In the eight combinations, the difference of mean difficulty was not statistically significant (*p* value >.05). Classifying the item difficulties into eight levels corresponding to the eight combinations of three factors was probably too fine-grained for a small number of samples. Testing the four regrouped combinations (based on the number of “high” factors), the result showed a statistically significant difference for item difficulty (*p* value <.05 level). This result is encouraging because it means that these factors did affect the item difficulty, which is proved by the fact that the items created with more “high” factors are more difficult than those with fewer “high” factors.

We next analysed how each factor contributes to the item difficulty using the three-way ANOVA. Among the three factors, DWD contributed the most to the item difficulty, followed by SIM and RPD.

However, this tendency is not retained when the same analysis was conducted with different proficiency-based groups of test takers (the third research question). For the high-proficiency group, SIM had the largest effect on item difficulty, suggesting that for this group, they were more likely to get confused when the correct answer was similar to the distractors. As for the middle-proficiency group, the overall tendency was retained; DWD had the largest influence, followed by SIM and RPD. The tendencies for both high and middle proficiency group suggest that RPD has the least effect on the item difficulty. One possible explanation is the test takers in these groups likely did not always read the reading passage to answer the vocabulary questions in the experiment. This finding, however, does not hold for the low proficiency group, where the item difficulty is influenced more by DWD, followed by RPD and SIM. This suggests that for the test takers with lower proficiency, RPD impacts on the item difficulty, in contrast to the other two groups. The test takers in this group probably read the reading passage to answer the questions.

The present study is the first step toward integrating the automatic question generation system with a computerised adaptive test. This study shows that item difficulty can indeed be controlled intrinsically by controlling the question components, thus suggesting the possibility of generating items automatically on-the-fly during the administration of a computerised adaptive test. The next step is to integrate the automatic question generation system with the item difficulty control into the CAT, moving toward a fully automatic system for administrating tests.

## Footnotes

1 Other parameters include item discrimination and guessing parameter.

2 A fill-in-the-blank question.

3 A test where some fraction of words have been removed from a text (gap) and the test taker is supposed to fill those gaps.

4 In the automatic question generation system used in this study, the website for the sources of the reading passage is decided before the system generates the questions.

5 The scores were calculated using the textstat package (version 0.3.1) in Python, available at https://pypi.python.org/pypi/textstat.

6 Brown, ABC, Genesis, Webtext, Inaugural and Gutenberg corpora are used, available at (http://www.nltk.org/nltk/_data/).

7 Stopwords corpus from NLTK (http://www.nltk.org/nltk/_data/).

8 We divided the students into three groups, following the standardised test score explanation, e.g. TOEFL https://www.ets.org/toefl/ibt/scores/understand/.

9 Pearson correlation is used (calculated with R) since our data follows normal distribution.

10
https://www.r-project.org.

11
http://mayekawa.in.coocan.jp/Rpackages/HTML_help/packages.html.

12 Function “shapiro.test” in R.

13 Function “leveneTest” in R.
